# Histopathology of Hidradenitis Suppurativa: A Systematic Review

**DOI:** 10.3390/dermatopathology9030029

**Published:** 2022-07-14

**Authors:** Shane David Basil Smith, Ginette A. Okoye, Olayemi Sokumbi

**Affiliations:** 1 Department of Pathology, The George Washington University, Washington, DC 20037, USA; 2 Department of Dermatology, College of Medicine, Howard University, Washington, DC 20059, USA; ginette.okoye@howard.edu; 3 Department of Dermatology, Mayo Clinic, Jacksonville, FL 32224, USA; sokumbi.olayemi@mayo.edu; 4 Department of Laboratory Medicine & Pathology, Mayo Clinic, Jacksonville, FL 32224, USA

**Keywords:** hidradenitis suppurativa, acne inversa, follicular occlusion, dermatopathology

## Abstract

Hidradenitis suppurativa (HS) is a chronic inflammatory scarring disease felt to be related to occlusion of the hair follicle unit in intertriginous areas. We perform a systematic review on HS histopathology to evaluate current knowledge and discuss future directions. PubMed and Scopus databases were searched for relevant articles published from January 1985 to January 2021 that discussed the pathology of HS. Additional articles were identified by hand-searching, which entailed manually scanning selected journals. A total of 355 citations were identified in the primary search within the main databases. Two hundred and seventy-nine articles were excluded after a review of titles, abstracts, and duplicates. Sixty-one studies did not meet the inclusion criteria or were found to be duplicates, resulting in a total of 15 articles for analysis. Three articles were hand-searched. This comprehensive systematic review of the histopathology of HS confirms a high prevalence of follicular occlusion, follicular hyperkeratosis, and hyperplasia of the follicular epithelium. These findings support the central role of follicular occlusion in the development and progression of HS while providing a potential path to directing therapeutics against follicular occlusion.

## 1. Introduction

Hidradenitis suppurativa (HS) is a chronic inflammatory scarring disease thought to involve occlusion of the hair follicle unit in intertriginous and anogenital areas [1,2]. The diagnosis is made clinically by the presence of recurrent abscesses, tunnels and scars in the typical anatomic regions. The prevalence of HS ranges from 0.03% to 4%, with pubertal onset and a female predilection [3]. HS may be associated with cigarette smoking, obesity, and inflammatory bowel disease [4], while a small percentage is associated with genetic mutations [5,6].

HS was initially considered an inflammatory process of sweat glands. Although the pathogenesis of HS is unclear, it is thought to be a disorder of the hair follicle [7]. HS is considered by many to have non-specific histopathologic features and at times is indistinguishable from other inflammatory conditions. Moreover, the histopathology of HS is not well-characterized and varies depending on the stage of the disease. In the early stages, prior to the establishment of lesions, there may be follicular occlusion of hair follicles with dilation at the distal end. Following the rupture of the hair follicle, there is suppurative inflammation, consisting mainly of neutrophils, with subsequent involvement of mononuclear cells and multinucleated giant cells.

Although the amount of research on HS has exponentially increased, a comprehensive review of the characteristic histopathologic features of HS remains lacking. We sought to perform a systematic review on HS histopathology to evaluate the current literature.

## 2. Methods

### 2.1. Data Sources and Search Strategies

A comprehensive search of two databases from January 1985 to January 2021 for histopathology of HS in the English language was conducted. PubMed and Scopus were the primary databases. The search system was designed by a medical librarian. Other relevant articles were identified by hand-searching, which includes manually scanning select articles.

### 2.2. Inclusion Criteria

The search terms were “hidradenitis suppurativa” and “acne inversa” in the title or abstract. Reviews and references were eligible for consideration in this review if (1) the article focus was either histopathology, pathology of hidradenitis suppurativa or acne inversa or (2) the data from ongoing pathologic research of “hidradenitis suppurativa” and “acne inversa” were discussed and (3) the article quantified histological features. Studies providing these objectives were included in the review. Only published case reports or case series in peer-reviewed journals published in English were included in this review, with publication years ranging from January 1985 to January 2021. In most case reports or case series, the lesion size and the specific anatomic site were available. One reviewer assessed the remaining articles for inclusion.

## 3. Results

**Figure 1:**
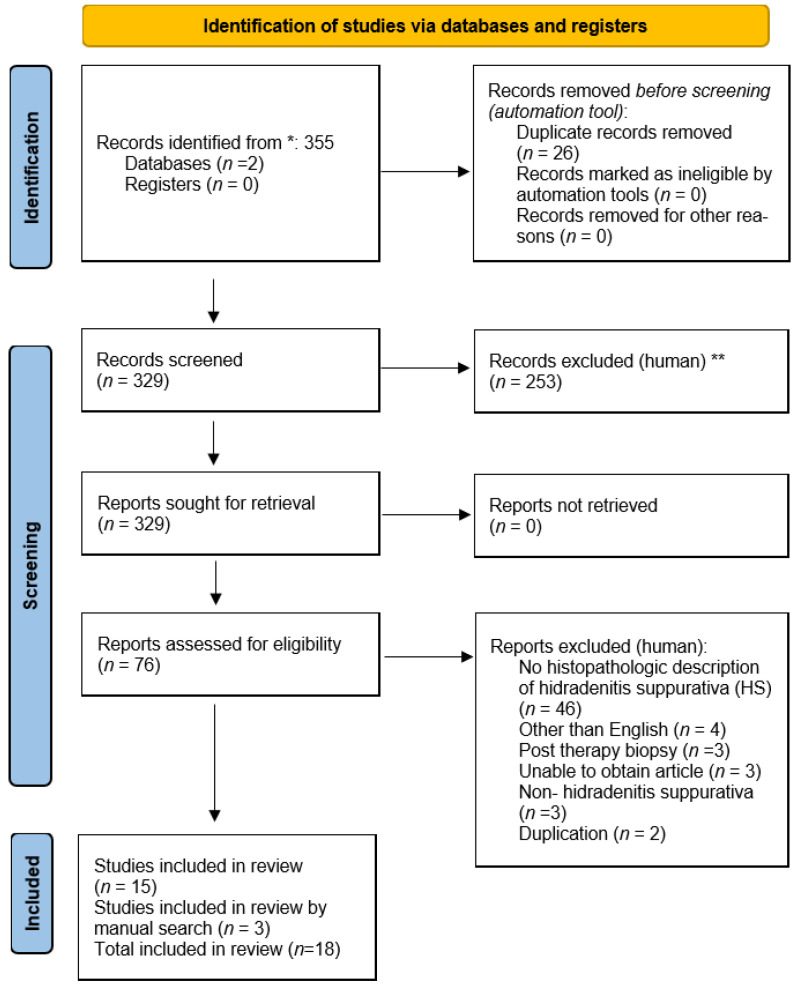
PRISMA flow diagram. (* Consider, if feasible to do so, reporting the number of records identified from each database or register searched (rather than the total number across all databases/registers). ** If automation tools were used, indicate how many records were excluded by a human and how many were excluded by automation tools) [26].

A total of 355 citations were identified in the initial search. Once adjusting for duplicates, 329 remained. Based on the review, 253 were excluded because they did not meet the inclusion criteria. After a thorough review of these remaining articles, 61 studies failed to meet the inclusion criteria or were found to be duplicates, resulting in a total of 15 articles for analysis and 3 additional articles were hand-searched (Figure 1) [8,9,10,11,12,13,14,15,16,17,18,19,20,21,22,23,24,25]. Of the 18 studies included in this review, all quantified and described the histopathology of HS.

## 4. Discussion

**Figure 2:**
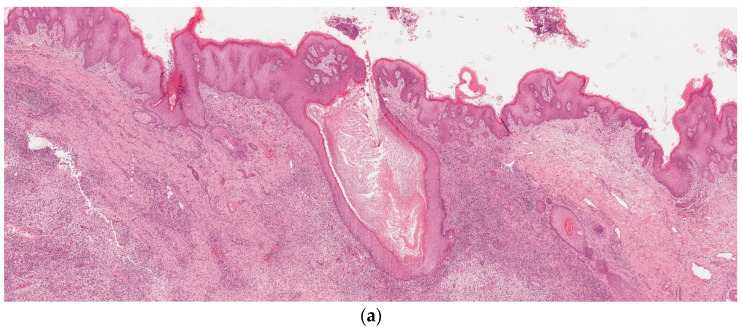

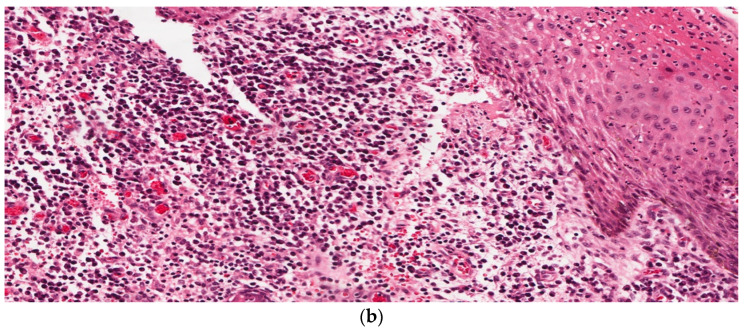
(**a**) Follicular plugging of the hair follicle-original magnification, 5× hematoxylin–eosin staining. (**b**) Mixed inflammatory infiltrates with a preponderance of plasma cells—original magnification, 20× hematoxylin–eosin staining.

The race to find effective therapeutic targets in the management of HS has accelerated in recent years. In addition to understanding the cytokine milieu of HS, the understanding of microscopic alterations in the disease is also of paramount importance. We performed a systematic review of HS in both older and contemporary studies to discuss histopathologic alterations identified in this complicated disease. We found that follicular occlusion (Figure 2a), follicular hyperkeratosis, hyperplasia of follicular epithelium, epidermal psoriasiform hyperplasia, and perifolliculitis were among the most common reported histopathologic features [8,17,21,24].

Interestingly, in the study by Attanoos et al. [21], only 25% and 8.4% of the specimen examined reported foreign body granulomas and epithelioid granulomas, respectively. Our review showed that the occurrence of foreign body granulomas and epithelioid granulomas are relatively uncommon in HS and the possibility of other granulomatous processes, such as Crohn’s disease, sarcoidosis or an infectious etiology (e.g., mycobacterium tuberculosis) should be considered [21]. In differentiating the granulomas present in cutaneous Crohn’s disease from HS, the utility of pan-keratin antibodies has been proposed to demonstrate the keratin remnants derived from the follicular cyst rupture present in HS and absent in Crohn’s disease [27]. Ancillary studies, including immunohistochemical stains (IHC), should be used judiciously in practice, as they may aid in differentiating mimics. Boar et al. characterized the inflammatory infiltrate of HS by using IHC, demonstrating that the infiltrate consists of cells positive for CD3, CD4, CD8, CD68, and CD79 with a CD4:CD8 ratio of 2.1:1 [20]. In the proper clinical context, special stains such as the Ziehl–Neelsen stain may be necessary [21].

The findings on the incidence of follicular occlusion, follicular hyperkeratosis, hyperplasia of follicular epithelium, and perifolliculitis after reviewing 18 studies (796 specimens) support the notion that hidradenitis suppurativa is a disorder of the hair follicle. Despite our findings, there is an absence of detailed histopathologic features of HS in the literature. Although follicular occlusion was not discussed, at least one article briefly mentioned the variation of epidermal changes, including infrequently observed parakeratosis [22]. Additionally, it is not uncommon for hyperplasia of the follicular epithelium to occur in the infundibular region [8].

Our review supports that there is no significant direct relationship between HS and sweat glands [17,24]. Instead, the sweat gland may be an infrequent “innocent bystander”, primarily involved due to the anatomical distribution of HS [17,24]. Although the involvement of sweat glands is relatively low, when involved, eccrine glands are predominantly involved [14,17,24]. There are two reasons for the higher frequency of eccrine gland involvement. First, eccrine glands may be quantitively more plentiful than apocrine glands due to limited sampling. Secondly, the increased involvement is conceivably due to the interaction of the dysfunctional inflammatory response to non-specific stimuli from eccrine glands. The former is more feasible and is most likely.

One recent study that examined HS histopathologic changes reported eosinophils in 10 of 16 lesional specimens examined [19]. Two additional studies also reported eosinophils, although few [13,17]. These findings were not consistent with previous studies, which did not report eosinophils in excision specimens. The lack of eosinophils, however, may be due to under-reporting in other studies.

**Figure 3:**
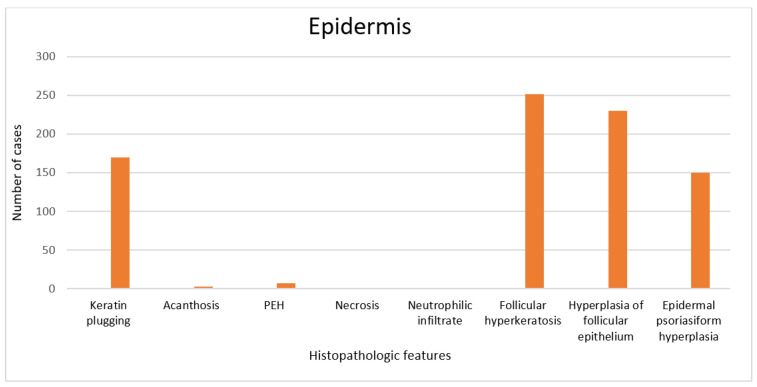
Histopathologic features of the epidermis from 796 samples (18 articles). Pseudoepitheliomatous hyperplasia—PEH.

**Figure 4:**
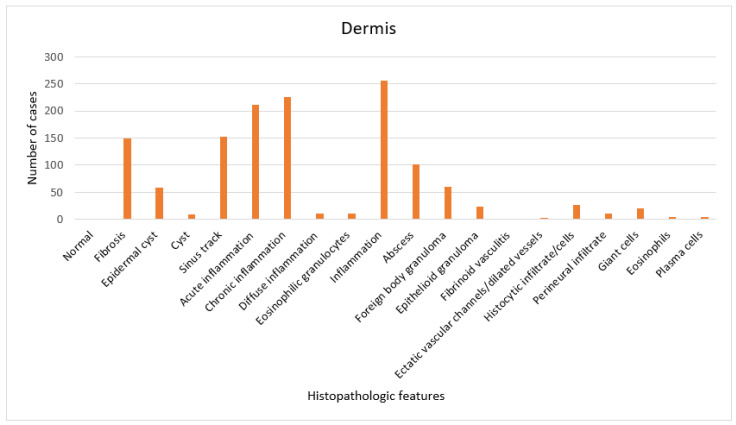
Histopathologic features of the dermis from 796 samples (18 articles).

Follicular occlusion has been considered the main histopathologic change in HS [5], and the literature supports the hypothesis that follicular occlusion plays a vital role in disease pathogenesis. This alteration is likely the trigger of the cyclic inflammatory process in the background of a genetic predisposition. Our review also reveals that perifolliculitis occurs at a relatively high frequency similar to hyperplasia of the follicular epithelium and follicular hyperkeratosis, 68%, 80%, and 89%, respectively [8]. In this self-sustaining circuit [7], the authors hypothesize that cytokine release cannot occur without follicular occlusion and vice versa. Follicular occlusion is expected to be directly proportional to perifolliculitis in this circuit, and our review showed they were directly proportional, supporting the important relationship between follicular occlusion and perifolliculitis [8]. Other findings in the epidermis and dermis include acute and chronic inflammation, plasmocytic infiltrate (Figure 2b), abscess, tunnels, cyst, fibrosis, pseudoeptheliomatous hyperplasia (PEH), and epidermal psoriasiform hyperplasia (EPH), which are not uncommonly seen (Figure 3 and Figure 4) [7,8,9,10,11,12,13,14,15,16,17,18,19,20,21,22,23,24,25].

Common mimics of HS include chronic deep folliculitis, Langerhans cell histiocytosis (LCH), Ref. [28] folliculotrophic mycosis fungoides (FMF), Ref. [29] abscess, follicular rupture, pyoderma gangrenosum, and Crohn’s disease. While abscess and chronic deep folliculitis may be challenging mimics, LCH and FMF should not be. LCH will characteristically have large cells with eosinophilic cytoplasm, and coffee bean elongated nuclei. FMF, on the other hand, will show atypical lymphocytic infiltrate with an aberrant immunophenotypic profile.

## 5. Conclusions

To our knowledge, this review is the largest systematic review on HS histopathology to date. However, there were several limitations. We only included studies that were in English, and the histopathologic classifications differed between studies making it challenging to draw comparisons. The pre-operative protocols varied between reports and, as a result, may impact the type of specimen examined. Additionally, the authors had minimal information about treatments given to patients prior to excision.

This systematic review confirms the involvement of follicular occlusion in HS and supports the need for therapeutic agents which inhibit the follicular plugging and/or normalize follicular keratinocyte growth and maturation. Developing and optimizing therapies can come to fruition through large prospective studies monitoring patients by utilizing objective clinical and histopathologic parameters. Through continued research incorporating pathology, we may optimize therapeutics, identify new targets, and improve the quality of life of HS patients.

## Data Availability

All data was referenced in this article.
